# Future Time Perspective Connectedness to a Career: The Contextual Effects of Classroom Knowledge Building

**DOI:** 10.5334/pb.282

**Published:** 2016-07-13

**Authors:** Jenefer Husman, Jonathan C. Hilpert, Sarah K. Brem

**Affiliations:** 1 Department of Education Studies, College of Education, 5277 University of Oregon, Eugene, US; 2 Department of Curriculum Foundations and Reading, Georgia Southern University, US; 3 Arizona State University, US

**Keywords:** Future Time Perspective, Self-Regulated learning, Multi-Level modeling

## Abstract

Professor Willy Lens has provided inspiration through his scholarship and mentorship for research in Future Time Perspective (FTP) theory. The traditional conceptualization of FTP consists of hierarchically organized psychological constructs that define individual differences in perceptions of the future across varying levels of specificity. The levels of specificity create a nested variable structure that is often described in a top-down fashion, from domain-general to context-specific. In the current study, relations among measures of *connectedness*, an FTP construct regarding concern for and planfulness about the future, are examined at three levels of specificity: domain-general, domain-specific, and context-specific. We examine interactions between domain-specific and domain-general levels of FTP. A sample of 3962 undergraduate engineering majors (mean age 20) from a large research university in the southwestern United States of America were surveyed. Hierarchical linear modeling was used to examine the hypothesis that aggregate classroom levels of student knowledge building moderate relations in the nested connectedness variable structure. At the student level of analysis measures of students’ domain-general, domain-specific, and context-specific connectedness were significantly and positively related. At the classroom level of analysis, results indicated that higher levels of aggregate classroom knowledge building shifted the direction of relations suggesting that in more engaging classroom contexts perceived value of learning for reaching a future goal may shape how students plan for future careers (domain-specific FTP). Implications for FTP theory are discussed.

For almost 40 years, Professor Willy Lens spearheaded the investigation of time perspective in academic contexts, arguing that student success is influenced by their ability to understand the value of their actions in the context of their past, present, and future (Lens, 1975, 1986; Lens, Paixão, Herrera, & Grobler, 2012; Nuttin & Lens, 1985, Phalet, Andriessen, & Lens, 2004). It was the inspiration Willy provided, through both his scholarship and tireless mentorship, that set us on a career-long course to contribute to the understanding of Future Time Perspective (FTP), or the degree to which a person integrates the chronological future into the present when setting and pursuing goals (Husman & Lens, 1999).

Time perspective has been examined at different levels of specificity: as a domain-general trait-like variable that is relatively stable (Harber, Zimbardo, & Boyd, 2003), as a domain-specific variable which may shift depending on the life domain under consideration (i.e., one may have a future orientation when thinking about family life, and a present orientation when thinking about academic life; Peetsma & van der Veen, 2011), and as a way to frame specific learning contexts, set of tasks, or content (Husman & Lens, 1999). Each level is conceptualized as having an independent yet interrelated influence on motivation in the present (Hilpert, et al., 2012). Although prior work has primarily focused on the motivational and behavioral outcomes of time perspective (see Husman et al., 2015) here we consider domain-specific time perspective regarding a future career as an outcome rather than as an explanatory variable. Typically FTP is framed from a top-down perspective: domain-general traits like time perspective contribute to individual’s time perspective within specific life domains (e.g., career or family), which in-turn relate to their perceptions of the specific relations between activities and learning content (i.e., calculus) and their future goals (e.g., Jung, Park, & Rie, 2015; Savickas, Silling, & Schwartz, 1984; Strauss, Griffin, & Parker, 2012). This direction of influence, however, is not the only possible one. In this study we propose an exploration of a more complex model of time perspective, one which considers domain the outcome variable and examines the interactions between students’ classroom experiences and their time perspective as an individual difference.

## Future Time Perspective: From Domain-general to Context-specific

Time perspective theory provides a framework for psychologists to study the cognitive and motivational constructs that underlie human understanding of their life activities in terms of the past, the present, and, in the area of FTP, the future (Carstensen, Isaacowitz, & Charles, 1999; Lens et al., 2012). Typically these constructs are represented in a nested fashion, with domain-general perceptions positioned as predictors of domain-specific and context-specific perceptions (e.g., Eren, 2012; Greene, Miller, Crowson, Duke, & Akey, 2004; Simons, Vansteenkiste, Lens, & Lacante, 2004). For example, in this special issue Fryer and his colleagues interpret their findings, the positive relationship between distal goals and motivation, through a discussion of students’ general future orientation (Fryer, Van den Broeck, Ginns, & Nakao, 2016).

Domain-general FTP constructs are conceptualized as slow to change orientations that develop throughout childhood and have achieved a state of equilibration, or the notion that some people have a tendency to think about the future more often than others, while others think more about the past or present (Zimbardo & Boyd, 1999). In classroom contexts, other researchers have focused on the way students value specific classes or content, considering the implications of learning specific content (e.g., calculus) for long term future goals (e.g., Malka & Covington, 2005; Tabachnick, Miller, & Relyea, 2008). Additionally, researchers have argued for the importance of considering FTP at the level of life domains (Seginer & Halabi-Kheir, 1998; Seginer & Shoyer, 2012). At this level, FTP is specific to the development of imagined futures within leisure, professional and career trajectories, social relationships, and so on (Hilpert, Husman, & Carrion, 2014; Peetsma & Stouthard, 1999).

Future time perspective research has provided support for the top-down nature of time perspective models (Zaleski, 1987). Researchers have provided evidence that individuals’ general orientation toward the future can influence the development of their thoughts about their personal futures within particular domains, creating a mental context for thinking about present contexts (Hilpert et al., 2012; Husman & Lens, 1999; Malka & Covington, 2005; Seginer, 2008; Zimbardo & Boyd, 2015). Utilizing time perspective surveys (e.g., Zimbardo & Boyd, 1999) researchers have demonstrated relations between trait-like FTP and a varieties of risk-taking behaviors (e.g., drug use Keough, Zimbardo, & Boyd, 1999), types of personal goals held (e.g., knowledge related or socio-emotional; Lang & Carstensen, 2002), and self-regulated learning (Gutiérrez-Braojos, 2015; de Bilde, et al., 2010) and career choice (Eren & Tezel, 2010). Utilizing survey methods we have explored the relationship between domain-general FTP, perceptions of instrumentality, and students’ use of learning strategies (Hilpert et al., 2012). In all of these studies FTP is represented as the predictor and behaviors are the outcome.

Although many researchers use domain-general and domain-specific constructs as predictors of context-specific phenomena, research suggests these perceptions can be altered by domain-specific experiences such as critical life events (i.e., combat experience) or direct therapy (Holman & Silver, 2005; Ferrari, & Díaz-Morales, 2007; Zimbardo, Sword, & Sword, 2012). Within educational settings there is evidence that learning contexts can influence students’ future academic goals and plans (Oyserman, Terry, & Bybee, 2002). Utilizing experimental designs, Destin and his colleagues have consistently demonstrated interactions between the influence of students’ developing perceptions of the likelihood of achieving a possible future and the effect of that possible self on behavior (e.g., Destin, 2016; Oyserman & Destin, 2013). These studies have led us to consider the need to test more complex models of time perspective than the typically discussed the top-down model of time perspective (Hilpert, et al., 2012; Husman & Lens, 1999; Nuttin & Lens, 1985). In this study we place domain-specific FTP as the primary outcome variable and examine the interactions between the domain-general, domain-specific, and classroom context factors. This design will allow us to consider both a top-down and bottom-up model of FTP.

## Connectedness: Multiple Levels of Analysis

Connectedness is a cognitive aspect of FTP which focuses specifically on the extent to which individuals plan for the future (Husman & Shell, 2008) that can be construed across multiple levels of generality. *Domain-general connectedness* is a psychological characteristic describing the ways that humans represent their personal future (Hilpert, et. al., 2012). Researchers have used different terms to describe this aspect of time perspective in their self-report measures such as concern for future consequences (Strathman et al., 1994) and future orientation (Gjesme, 1983), or as part of a general measure of time perspective (Zimbardo & Boyd, 1999). Each of these conceptualizations have demonstrated a relationship between connectedness and behavioral outcomes in the present. For example, Walker and Tracey (2012), utilizing Husman and Shell’s (2008) time perspective scale, demonstrated that individual differences in connectedness to the future is positively related to students’ confidence in their ability to make career decisions, a decrease in their anxiety about choosing and committing to a career, and decreased sense of being underprepared.

*Career connectedness* is a domain-specific construct that describes the general tendency to plan for a future career. Emerging adults, in particular, engage in a considerable amount of planning for the future and considering connections between specific domains of their lives and the decisions they are making in the present. One central focus of their planning behavior focuses on future possible careers (Salmela-Aro & Nurmi, 1997; Schwartz, Côté, & Arnett, 2005). Husman, Duggan, and Fishman (2014) modified Husman and Shell’s connectedness subscale to represent the general tendency to think about and plan for a desired future career. This revised scale has demonstrated strong reliability and structural validity as evidenced by confirmatory factor analysis (Husman, et al., 2012). Prior research has demonstrated that although career connectedness is significantly and positively correlated with domain-general connectedness, structural models provide evidence for two independent constructs (Husman, Fishman, Nelson, & Hilpert, 2015). The effectiveness of this measure has been demonstrated in two career domains, engineering and teaching (Husman, et al., 2012), suggesting that career connectedness carries unique information that accounts for variation in students’ strategic learning and motivation over and above domain-general connectedness (Husman et al., 2015).

Student connectedness to the future with respect to context-specific activities has been operationalized as *perceptions of instrumentality* (Kover, & Worrell, 2010; Simons, Dewitte, & Lens, 2004). Instrumentality is defined as a person’s perception of how useful set of present tasks or learning specific content is for a desired future goal (Raynor, 1981; Fryer, Van den Broeck, Ginns, & Nakao, 2016). Students’ positive perception of instrumentality is related to learning gains in the classroom (Malka & Covington, 2005; Tabachnick, Miller, & Relyea, 2008). Top-down FTP models suggest that students with strong domain-general and career connectedness can more easily see the instrumentality of their current class activities for distant future goals (Hilpert et al. 2012; Husman & Lens, 1999) and are more likely to be motivated by those connections (Zaleski, 1987; Fryer, Van den Broeck, Ginns, & Nakao, 2016). Students’ with strong connectedness seek out information they can use to judge the value of courses (Lens, Paixão, Herrera, Grobler, 2012). Although perceptions of instrumentality are influenced by students’ FTP there is also evidence that students’ perceptions of instrumentality are influenced by classroom characteristics (Greene & DeBacker, 2004).

All three levels of specificity are part of the model psychologists use when describing the influence of future thinking on behavior (Shell & Husman, 2001). All three levels work together to support adaptive planning behavior (Oyserman, Bybee, Terry, & Hart-Johnson, 2004). For example, university students focused on the extended future (domain-general) are more likely to have well developed imagined possible career selves (domain), and therefore can better evaluate decisions about which will be their academic major. Their vision of their future, shaped by their domain-general and domain-specific FTP will frame their understanding about which classes will serve as a means to attain their future self; the connection they make between the class content and the future ideal self will influence their motivation for learning in that course (Packard & Nguyen, 2003). This is a top-down approach to examining FTP (domain–general → domain → context → outcome).

To examine the interactions between all levels of specificity of time perspective we tested a nested model composed of connectedness variables operationalized as domain-general connectedness, career connectedness, and instrumentality (see 1). We consider domain-specific career connectedness as the outcome lying between domain-general and context-specific FTP (domain-general → domain-specific ← context); in that way predicting domain-specific FTP by domain-general FTP would test the top-down effects, whereas predicting domain-specific FTP by context-specific FTP would test the bottom-up effect.

**Figure 1:**
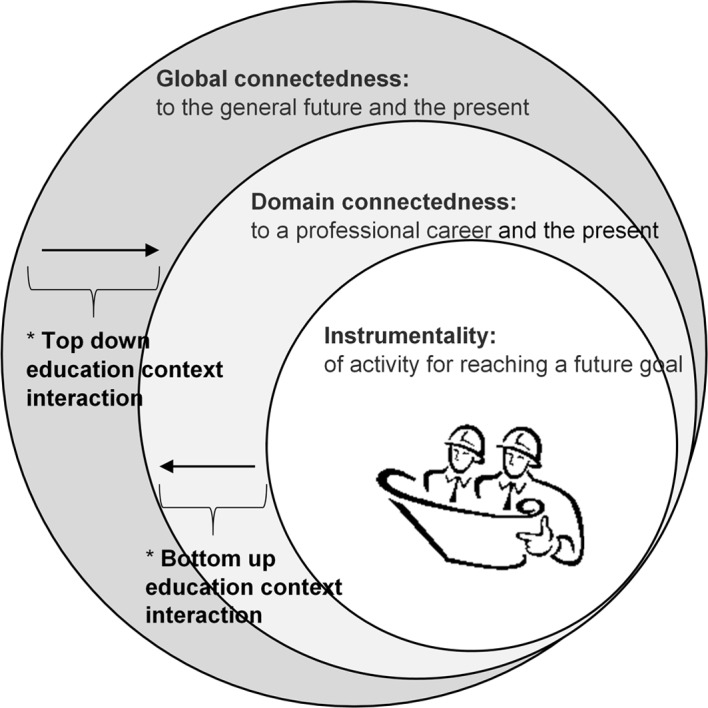
Gobal to instrumental future time perspective variable sequence. *Note.* The figure represents the nested structure of the FTP constructs. Educational context interaction refers to how a) the class level context and b) enrollment in increasingly rigorous courses impacts how global connectedness and instrumentality influence connectedness to a career within a life domain.

The exploration of relationship between levels of FTP may provide researchers with valuable insight into mechanisms of effect with implications for both research and practice. We bounded our study to a particular academic domain (i.e. engineering majors) and argue that the FTP hierarchical variable structure will vary as a function of the specific classroom context. Below we describe the classroom context of our study using Bereiter and Scardamalia’s (2014) theory of knowledge building communities.

## Classroom Context and Student Knowledge Building

In the current study, we examined connectedness within a sample of university students studying to become engineers. Within the context of engineering education researchers argue that the ability to produce new knowledge in response to technological problems is a key educational outcome (Siddique, Ling, Roberson, Xu, & Geng, 2013). In the United States, the university level represents the first time that students are expected to take primary responsibility for their learning (Tinto, 1993). University faculty expect that students will go beyond the facts that are presented to them by an in-depth study of a topic that goes beyond recall learning. This requires connecting new information to existing knowledge and the integration of knowledge across topics and domains. Knowledge building, a construct initially presented by Carl Bereiter and Marlene Scardamalia (e.g., 2014) represents this type of deep learning. Knowledge building has been described as both an individual strategy (Nelson et al., 2015) and a characteristic of a learning community (Scardamalia & Bereiter, 1999). Knowledge building as a feature of a classroom is the production of new knowledge through setting common goals, engaging in group dialogue, and synthesizing ideas through collective inquiry (Bereiter & Scardamalia, 2014). Shell and colleagues (2005) found that students in classrooms classified as highly collaborative communities self-reported higher levels of strategic self-regulation and knowledge building than those in comparison groups. In this study we focus on knowledge building at the classroom rather than individual level.

## Goals of the Current Research

Many examples of FTP research representing the top down influence of domain-general time perspective on domain and context-specific outcomes exist (Gutiérrez-Braojos, 2013; Hilpert et al, 2012; Husman & Lens, 1999; Luyckx et al., 2010; Zimbardo & Boyd, 1999). We argue that this hierarchical model is made more complex by the possibility that classroom experiences can shape imagined futures. Levels of time perspective may work in both top-down and bottom-up fashion, and classroom contexts, such as the support of knowledge building, may shift the direction of these relations. This complexity can be examined by testing how educational contexts interact with FTP at different levels of generality (i.e., domain and domain-general). To this end we used hierarchical linear modeling to 1) to replicate the expected relations between each level of connectedness measured (domain-general, domain, context-specific), 2) examine the variance accounted for in career connectedness by aggregate classroom knowledge building, domain-general connectedness, and perceived instrumentality and 3) examine the effect of aggregate level classroom knowledge building on the strength and direction of these relations. Specifically, we address the following research questions:

1. RQ1: Does the covariation among the study variables indicate that aggregate classroom knowledge building, domain-general connectedness, and perceived instrumentality positively and significantly predict career connectedness?
  - RQ1a: how much of the variation in each of the variables is attributable to the individual and the classroom level?
  - RQ1b: how much of the variation in career connectedness at the student level is accounted for by the knowledge building as a classroom characteristic?
  - RQ1c: how much of the variation in career connectedness at the student level is accounted for by domain-general connectedness and perceived instrumentality?
2. RQ2: Does the relationship between a) perceived instrumentality and career connectedness, and between b) domain-general connectedness and career connectedness in the domain-general to instrumental sequence depend upon the amount of classroom knowledge building?
  - RQ2a: Is the domain-general connectedness effect on career connectedness larger in classes with higher aggregate levels of knowledge building?
  - RQ2b: Is the perceived instrumentality effect on career connectedness larger in classes with higher aggregate levels of knowledge building?

## Method

### Participants

Study participants were 3962 engineering majors enrolled at a large research university in the southwestern United States of America. In the sample, 84% were male. All participants had declared an engineering major. The average participant age at the time of data collection was 20 years old (*SD* = 3.4 years).

### Measures

**Domain-general Connectedness (GC).** The Future Time Perspective Scale (FTPS, Husman & Shell, 2008) was administered to assess domain-general connectedness. Example items from the six-item connectedness subscale are, “What might happen in the long run should not be a big consideration in making decisions now,” and, “I should be taking steps today to realize future goals.” The subscale contains both positively and negatively worded items, and participants responded to a Likert-type scale ranging from 1 (strongly disagree) to 5 (strongly agree).

**Career Connectedness (CC).** An adapted version of the FTPS connectedness subscale (Husman & Shell, 2008) was administered to assess participants’ motivation to seek out and plan for a future career in engineering. An example item from the six-item scale is, “It is important to have goals for where one wants to be in five or ten years as an engineer.” The subscale contains both positively and negatively worded items, and participants responded to a Likert-type scale ranging from 1 (strongly disagree) to 5 (strongly agree).

**Perceived Instrumentality (PI).** The perceived instrumentality scale (Husman et al., 2004) was administered to assess endogenous instrumentality for learning course material to attain their future goals. An example items from the four-item scale is, “I will use the information I learn in (course selected) in my future career.” The subscale contains both positively and negatively worded items, and participants responded on a Likert-type scale ranging from 1 (strongly disagree) to 5 (strongly agree).

**Student Perceptions of Classroom Knowledge-building (KB).** The student perceptions of classroom knowledge-building subscale (Shell et al., 2005) was administered to assess knowledge construction through cognitive elaboration in the classroom. An example knowledge-building item from the eight-item scale is, “Whenever I learn something new in this class, I try to tie it to other facts and ideas that I already know.” The students responded on a Likert-type scale ranging from 1 (almost never) to 5 (almost always).

Reliability of each measure was strong. Cronbach’s alpha was calculated for each study variable. Results suggested that student responses to the domain-general connectedness (α = .76), career connectedness (α = .82), perceived instrumentality (α = .88), and knowledge building items (α = .90) items were internally consistent.

### Procedures

Data collection spanned eight semesters from fall of 2007 to spring of 2010. Researchers contacted the course instructors for permission to visit their classes and invite students to complete an online survey. Participants received a small monetary incentive for participation. Students were recruited from 249 classes (e.g., an individual class of 40 students).

**Figure 2:**
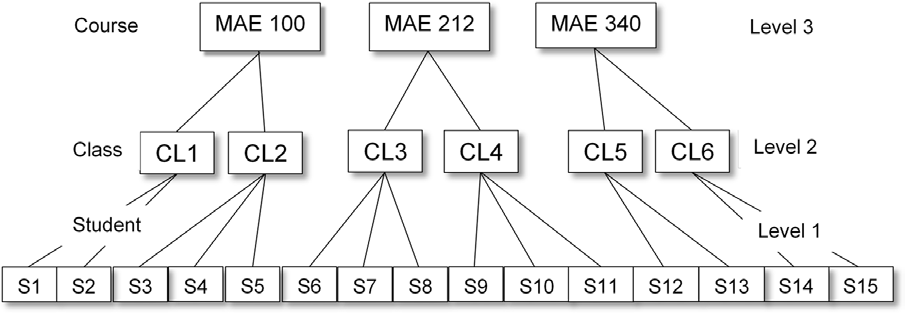
Sehematic representation of three level, nested data structure. *Note.* MAE 100 = example Mechanical and Aerospace Engineering (MAE) course in the data set. CL1 = example MAE 100 class in the data set. S1 = example student in MAE 100 CL 1 in the data set.

The data collection produced a two level, nested data structure with students nested within classes (See Figure 2). Data were cleaned and coded and assumptions checking was conducted. Data were inspected for outliers and missing survey item data were handled using multiple imputation (IBM, 2012). The number of missing values for the subscale items ranged from 50 to 281, and Little’s MCAR test using Expectation Maximization (EM) was not significant, Chi-Square = 4.94; *df* = 3, *p* = .17 indicating missing values were likely missing at random. We used IBM SPSS 21 to conduct a multiple imputation (fully conditional MCMC, linear regression including two-way interactions, five imputations), using existing item values and variables as predictors to estimate missing values. This approach maintains the overall variability in the population while preserving relations with other variables.

### Analysis

Within and between group variance components for classes and courses were calculated to answer research question 1a. Then, data were analyzed using HLM 7 for windows and the accompanying user guide (Raudenbush, Bryk, Cheong, Congdon, & Toit, 2011). Explanatory variables were centered around the group mean. We began our model building process by examining the variance component for the outcome variable career connectedness, and then adding predictor variables. To answer research question 1b we added classroom knowledge building at level-2 to create a random intercept model with a level-2 predictor. See equation (1).

1 \[
C{C_{ijk}} = {\gamma _{\it 000}} + {\gamma _{\it  010}}K{B_{jk}} + {r_{\it 0jk}} + {u_{\it 00k}} + {e_{ijk}}
\]

We continued our model building process in step-by-step fashion, systematically adding variables at levels of analysis (e.g. Hox, 2002) to analyze cross level interactions. We developed two models to complete the data analysis and answer our remaining research questions.

Specification of the level-1 random-regression coefficients model defined a set of level-1 coefficients to be computed for the level-2 units. This model allowed us to answer research question 1c. In the model, career connectedness is the outcome to be predicted by domain-general connectedness and perceived instrumentality. See equation (2).

2 \[
C{C_{ijk}} = {\gamma _{\it 000}} + {\gamma _{\it 100}}G{C_{ijk}} + {\gamma _{\it 200}}P{I_{ijk}} + {r_{\it 0jk}} + {u_{\it 00k}} + {e_{ijk}}
\]

Specification of the level-2 prediction model established each level-1 slope coefficients as an outcome variable. This model allowed us to answer research question 2a and 2b. The variation in the slopes are differences in strength of the relationship between a) domain-general connectedness and career connectedness and b) perceived instrumentality and career connectedness across aggregate levels of class knowledge building, or two way interactions. See equation (3).

3 \[
\begin{array}{l}
C{C_{ijk}} = {\gamma _{\it 000}} + {\gamma _{\it 100}}G{C_{ijk}} + {\gamma _{\it 110}}G{C_{ijk}}K{B_{jk}} + \\
{\gamma _{\it 200}}P{I_{ijk}} + {\gamma _{\it 210}}*P{I_{ijk}}K{B_{jk}} + {r_{\it 0jk}} + {u_{\it 00k}} + \\
{e_{ijk}}
\end{array}
\]

## Results

Descriptive statistics for the study variables were calculated and assumptions checking for univariate normality was conducted. Results suggested none of the study variables violated assumptions required for statistical testing (see Table 1), save for obvious concerns about clustering effects of students nested within classes. Analysis of the class group variance components indicated that 15.1% of the variance in knowledge building; 14.5% if the variation in perceived instrumentality, 13.8% of the variance in career connectedness, 12.4% of the variation in domain-general connectedness existed between the class groups.

**Table 1:** Descriptive Statistics and bivariate correlations for study variables.

	Min	Max	M	SD	Skew	Kurt	1.	2.	3.

1. Domain-general Connectedness	2.00	5.00	4.16	0.55	–0.53	–0.06			
2. Career Connectedness	1.33	5.00	4.12	0.57	–0.45	0.21	0.67		
3. Perceptions of Instrumentality	1.00	5.00	4.01	0.78	–1.02	1.54	0.21	0.31	
4. Knowledge Building	1.00	5.00	3.36	0.76	–0.23	0.23	0.14	0.22	0.49

*Note.* All correlations are significant at *p* < .01. Coefficients for KB are based on individual level scores for each student. In subsequent analyses, these scores are aggregated to the class level.

The random intercept model with a level-2 predictor was tested using knowledge building as the level two predictor of career connectedness. The regression coefficient relating aggregate classroom knowledge building to students’ career connectedness was positive and statistically significant (b = .18, *p* < .001). This demonstrates a significant positive relationship between a classroom’s focus on building knowledge and individual students’ career connectedness.

The level-1 random-regression coefficients model was tested using domain-general connectedness and perceived instrumentality as predictors of career connectedness. Model specification reduced the variance component for career connectedness by 46.3%. The regression coefficient relating students’ domain-general connectedness to students’ career connectedness was positive and statistically significant (b = .64, *p* < .001). The regression coefficient relating students’ perceived instrumentality to students’ career connectedness was also positive and statistically significant (b = .13, *p* < .001). Significant (both practically and statistically) variance in students’ career connectedness is accounted for by both domain-general connectedness and context-specific connectedness.

The level-2 slopes-as-outcomes model was tested with aggregate class knowledge building as a predictor of the variation in slopes for both variables at level-1. Model specification reduced the overall variance component for career connectedness by .06%. The cross-level interaction between students domain-general connectedness and class knowledge building was negative and statistically significant (b = –.16, *p* < .001). This finding suggests that the positive relation between students’ domain-general connectedness and students’ career connectedness (i.e., the model testing the top-down effects) was attenuated among students belonging to classrooms which were characterized by high knowledge building. The cross-level interaction between students perceived instrumentality and class knowledge building was positive and statistically significant (b = .11, *p* < .001). This finding suggests that the positive relation between students’ perceived instrumentality and students’ career connectedness (i.e., the model testing the bottom-up effects) was even stronger among students belonging to classrooms which were characterized by high knowledge building. Taken together the findings from the two cross-level interactions suggest that classroom contexts that support student knowledge building can shift the direction of the relationship within the nested variable structure from top-down, to bottom-up, where the perceived instrumentality of the activity shapes student connectedness to a future career. Classes where students engage in high levels of knowledge building strengthen the contribution of perceived instrumentality to career connectedness and weaken the relation of domain-general connectedness to planning for a future career.

## Discussion

This study provides a new window into the dynamic relationship between the multiple ways that FTP is constructed by students. Previous research suggested that the domain-general and domain-specific level of FTP frame student understanding of the instrumentality of a particular class and the motivation framing of a particular class is expected to influence students’ approaches to learning. Our findings indicate that the relation is not as simple. At the individual level, as expected, our measure of students’ domain-general, domain, and context-specific FTP were all positively related. When we considered students within specific class contexts, however, the direction and strength of these relations changed.

### The Future Time Perspective Model – Top-down and Bottom-up?

As expected, the regression coefficients relating students’ domain-general connectedness to students’ career connectedness was positive and statistically significant, as was the positive regression coefficient relating students’ perceived instrumentality to their career connectedness. These findings provide evidence that both domain-general and context-specific FTP variables play an important role in planning for future careers (Hilpert et al., 2014; Peetsma & van der Veen, 2011). Presented as such, the evidence implies that individual differences in domain-general connectedness to the future (i.e. Husman et al., 2015; Zimbardo & Boyd, 2015) and context-specific perceptions of instrumentality (Malka & Covington, 2005; Tabachnick, Miller, & Relyea, 2008) are both significant and positive predictors of career connecntedness.

However, the results of the current study complicate many top-down assumptions about future time perspective (Zimbardo & Boyd, 2015). The two way, cross-level interaction between students’ domain-general connectedness and knowledge building was negative and statistically significant, meaning the higher the aggregate class knowledge building, the less domain-general connectedness contributed to career connectedness. Additionally, the cross-level interaction between students perceived instrumentality and knowledge building was positive and statistically significant meaning the higher aggregate class knowledge building, the more perceived instrumentality contributed to career connectedness. These findings suggests that in classroom contexts where students are cognitively elaborating to produce knowledge, the connections they see between their present actions and their future careers may be driven from classroom activity, not from individual differences in the domain-general FTP of the student. These findings provide evidence that instructors may have the ability to build students’ career FTP through creation of knowledge building environments, regardless of whether students come to the class with strong FTP tendencies. These findings extend existing research which emphasized the importance of context-specific FTP on student learning (Fryer, Van den Broeck, Ginns, & Nakao, 2016; Simons, et al., 2004), suggesting that students’ career connectedness (domain level FTP) may be supported by the course’s instrumentality for future goals. These findings are an indication that the interaction of aggregate class level knowledge building and perceived instrumentality may have a bottom-up motivational influence on career connectedness and perhaps, by extension, identity development, moderating the contribution of individual differences in domain-general future perspective.

Connectedness to a future career may be influenced by classroom environments where cognitive elaboration is ongoing, implying the impact of perceived instrumentality is increasingly important to perceived connectedness to a career and domain-general connectedness detracts; whereas in environments where engagement is perfunctory, domain-general connectedness may be more central to the maintenance of staying connected to a career identify for motivational purposes. As university students are likely to experience classroom environments with varying levels of knowledge building, as well as other related variables such as boredom, engagement, and so on, the evidence presented here suggests both perceptions of instrumentality and domain-general connectedness may be important to the development and maintenance of students’ career connectedness, allowing students to adapt from context to context by maintaining a connection to the future.

### Limitations

This study suffers from the limitations common to survey cross sectional research studies. The analytic approach allows us to speculate on the possible bi-directionality of the influence of classroom contexts on career connectedness. The findings here are essentially correlational and cannot demonstrate direction of effect of the constructs we consider. Further longitudinal research examining the relationship of educational experiences overtime on the development of students’ FTP is required. Additional experimental research examining the influence of particular educational activities on the development of students’ career connectedness would also be informative. The findings in this study provide a justification for these efforts.

The implications of findings presented here for FTP theory are limited by the population the study was sampled from. University engineering students are a unique population. For example, in the United States, the overwhelming majority of engineering students are male (> 80%) and they are all taking a challenging course load. Examination of this population is important in and of itself, within the United States there is a significant need for highly educated engineers to enter the workforce, and others have recommended examining FTP within achievement settings (Mouratidis & Lens, 2015).

## Conclusion

The findings presented in current study leave us to speculate on the leverage points for supporting students’ perceptions of instrumentality and their career connectedness as the directionality of these relations is not determined. Our findings suggest that classroom environments which emphasize instrumentality and knowledge building strategies may optimize the impact of educational environments on career connectedness, and in less engaging environments students may adapt by relying on their domain-general plans for the future as a motivational frame for career planning. Additional research is needed to understand ways in which student learning within one classroom can support their perceptions of the instrumentality of particular content, the degree to which their understanding of the content can support their understanding of the particular career path, and the degree to which the career path can clarify persons imagined future. Lens and his colleagues (Lens, et al., 2012) have called for an expansion of this research into more emerging adult contexts, encouraging us to continue to examine family and social life domains, non-western contexts, and adult developmental contexts. Although some researchers have taken up these goals, examining the intersections of culture, classroom context, and time perspective (Bouffard, Bastin, & Lapierre, 1996; Jones & Leitner, 2015) and extending the life-domains to be considered (Herrera, 2010), we have a long way to go. It is clear that Lewin (1942) was on to something: we do live within a temporal space and Lens’ 40 years of consistent and high quality research has provided time perspective researchers with a solid foundation to take up his call and continue expanding that research for another 40.
